# Exploration of over the counter sales of antibiotics in community pharmacies of Addis Ababa, Ethiopia: pharmacy professionals’ perspective

**DOI:** 10.1186/s13756-016-0101-z

**Published:** 2016-01-29

**Authors:** Gebremedhin Beedemariam Gebretekle, Mirgissa Kaba Serbessa

**Affiliations:** School of Pharmacy, College of Health Sciences, Addis Ababa University, Addis Ababa, Ethiopia; School of Public Health, College of Health Sciences, Addis Ababa University, P .O. Box: 1176, Addis Ababa, Ethiopia

**Keywords:** Antibiotics, Over the counter, Nonprescription sale, Antibiotic resistance, Antimicrobial resistance, Community pharmacy, Addis Ababa, Ethiopia

## Abstract

**Background:**

Over the counter sale of antibiotics is a global problem and it is increasingly recognized as a source of antibiotic misuse and is believed to increase treatment costs, adverse effects of treatment and emergence of resistance. The increasing trend of over the counter sale of antibiotics in Ethiopia calls for exploration of why such dispensing is practiced. This study aims to explore reasons for over the counter sale of antibiotics in the community pharmacies of Addis Ababa, Ethiopia.

**Methods:**

A phenomenological qualitative study was conducted in five randomly selected community pharmacies of Addis Ababa. One pharmacy professional from each pharmacy were interviewed at the spot using semi-structured, open-ended interview checklist. Besides, observation of professionals’ dispensing practice was made for at least one hour in the same community pharmacies using an observation checklist. Findings were categorized into specific themes that were developed following the objectives. This was facilitated by use of OpenCode 3.6 software.

**Results:**

All participants pointed out that antibiotics were frequently dispensed without prescription and contend that the trend of such dispensing has been increasing. The findings indicated that the nonprescription sales of antibiotics were common for Amoxicillin, Ciprofloxacin and Cotrimoxazole. The poor, less educated and younger groups of the population were reported to frequently request antibiotics without prescription. The main reasons for nonprescription sale of antibiotics by pharmacy professionals were found to be related to pharmacy owner’s influence to maximize revenue, customer’s pressure, weak regulatory mechanism and professional conflicts of interest.

**Conclusion:**

The study shows that nonprescription sale of antibiotics was common practice at least in Addis Ababa. The main reasons for this malpractice were the need to maximize revenue and weak regulatory mechanism. Hence, strong regulatory enforcement and community awareness campaign is called for to limit nonprescription sale of antibiotics.

## Background

Despite the successful effects to control infectious diseases, the continuous decline of therapeutic effectiveness of antimicrobials due to among others increasing antimicrobial resistance has become a worldwide problem [1, 2]. Although globally antimicrobial resistance is an emerging threats to public health, the problem is more severe in developing countries like Ethiopia where the burden of infectious diseases is relatively greater and healthcare spending is low [3–7].

Drug resistance can be a natural phenomenon. Its proliferation, however, is attributed to multifaceted factors such as overuse and misuse of antibiotics [8–11]. Nonprescription sale of antibiotics is one of the major reasons to increasing antibiotic consumption which facilitates emergence of drug resistance [5, 12–14].

In Ethiopia, over the counter (OTC) sale of antibiotics at partial doses and without prescription are common practices, although the practice is not legal [15–25]. The misuse and overuse of antibiotics in Ethiopia coupled with weak regulatory mechanisms contributed to increasing antimicrobial resistance ranging from 0-100 % [26–28]. Hence, the aim of the study is to explore reasons for OTC sales of antibiotics without prescription. The findings from this study would help to develop strategies to promote prudent use of antibiotics in the community pharmacies.

## Methods

### Study design

A phenomenological qualitative study designed was followed. In-depth interview and observation were employed to generate information on pharmacy professionals’ antibiotic dispensing practice and why they sell antibiotics as OTC medicines. The study was conducted between August 24, 2015 and September 02, 2015.

### Background of the study area

The study was conducted in Addis Ababa, the diplomatic capital of African Union and capital city of Ethiopia [29]. According to Central Statistical Agency’s population projection, the population of Addis Ababa is 3.2 million of which 52.6 % are females [30]. Addis Ababa has a total of 308 pharmacies, 249 drug stores, 1 rural drug vendor, 759 clinics, 140 importers and 93 wholesalers of human medicine. Six of the 11 pharmaceutical industries in Ethiopia are also found in Addis Ababa [31].

### Data collection

A comprehensive list of community pharmacies in Addis Ababa was generated from Addis Ababa Food, Medicine and Health Care Administration and Control Authority (FMHACA). Addis Ababa has three types of community pharmacies or drug retail outlets namely: pharmacy, drug store/shop and rural drug vendor. This name difference, as listed from highest to lowest level, is indicative of the type of drugs they should dispense and thus the level of services they offer. Of these, the study targeted the highest level community pharmacies, pharmacy and drug store. The community pharmacies were randomly selected for the study one at a time and selection continued until information reached saturation, which was the case at four but one additional interview was made for confirmation.

The in-depth interviews were conducted at the spot with dispensing pharmacy professionals using semi-structured open-ended interview checklist with probing on prevailing dispensing practice. The interview questions were classified into two sections. The first section focused on personal and professional information such as the pharmacy professional’s age, work experience and educational background. The second section was designed to explore the professional’s view on the current antibiotic dispensing practice and reasons for OTC sales of antibiotics.

Two pharmacists with previous experience in qualitative methods of data collection were trained to carry out the interview. The average duration of the interview was seventy minutes, ranging from 55–80 min. In cases where interruption by customers is frequented interview has taken longer. Interviews were tape recorded and scribbles were also taken for expansion later. The interview was done in Amharic and any ambiguities raised from the interviewee were cleared at the time of the interview. In addition to the interview, observation of pharmacy professionals’ dispensing practice was done in the same pharmacies for additional one hour during a peak hour using a checklist developed for this purpose. Observation focused on the antibiotic dispensing practices, extent of OTC sales of antibiotics, the most frequently sold antibiotics and the type of customers who frequently request for nonprescription sale of antibiotics. Overall, observation helped us to triangulate with the findings of in-depth interview.

### Data management and analysis

A code book was prepared prior to data collection. Audio-recorded Amharic version of the interviews and observations were translated to English and complete transcripts of all interviews and observation note were prepared. The notes were intensively read and the raw data was categorized under pre-developed coded themes and sub themes. Four major themes were defined: antibiotic dispensing practices; characteristics of customers requesting for OTC sales of antibiotics; types of antibiotics frequently sold without prescription; and reasons for OTC sales of antibiotics. Within the perceived reasons for OTC sales of antibiotics, four major sub-themes were emerged again. The analysis was facilitated by OpenCode 3.6 software. To check the accuracy of the translation, one of the recordings was translated and transcribed by a bi-lingual pharmacist and compared with the primary work. In reporting the findings, codes were used to maintain anonymity of participants. Furthermore, findings of the study were communicated to couple of the study participants for authenticity of interpretations.

### Ethical considerations

Ethical clearance was obtained from School of Pharmacy, College of Health Sciences, Addis Ababa University and verbal consent was secured from each participant prior to the data collection.

## Results

A total of five in-depth interviews were done with pharmacy professionals from the community pharmacies in Addis Ababa. Three of the participants were pharmacists holding bachelor degree in pharmacy (B. Pharm) while the rest were druggists holding diploma in pharmacy. Their age range from 27–37 years. In terms of job experience, the participants had worked in community pharmacy for a range of 2 to 8 years.

### Antibiotics dispensing practice

All (*n* = 5) study participants agreed that the dispensing practice was not up to the standards set by the regulatory authority. The participants also mentioned that majority of the pharmacy professionals didn’t comply with the country’s law, professional code of ethics and regulatory guide of good dispensing practice. Non-prescription sale of antibiotics was recognized to be a growing public health problem by all participants. Majority (*n* = 4) of them believed the trend was of nonprescription sale as increasing. This was strengthened by one of the participant that:

*“….. Although we professionals know antibiotics are prescription only medicines that shouldn’t be dispensed without prescription, most of us failed to do so. Antibiotics are highly misused medicines and nonprescription sale of these medicines is increasing from time to time (CMP003).*

One of the participants however was uncertain on whether such practice is increasing or decreasing. He pointed out that *“the trend of nonprescription sale of antibiotic in each pharmacy seems slightly decreasing but there is an increase in the number of pharmacies dispensing antibiotics without prescription. Hence, cumulative effect of OTC sale of antibiotics might be higher or lower which calls for a wider comparative research (CMP001).”*

Although majority (*n* = 3) of the participants noted that the nonprescription sale was a common practice in most of the community pharmacies, all Kenema public or government pharmacies and very few private pharmacies were not victim of this irrational dispensing practice. Findings from the observation also indicated that the practice of OTC antibiotic dispensing was common practice in five of the community pharmacies. It was found that pharmacy professionals were seen selling antibiotics without prescription. This is further substantiated by one of the participants that;

*“…….if someone needs and asks to get antibiotic, s/he can get whatever antibiotics s/he needs from any pharmacy regardless of its level (generation). I mean the practice is not in line with what we know as a standard and such practice is becoming common. Yet, nothing has been done to control nonprescription sale of antibiotics (CMP002).”*

### Characteristics of customers requesting OTC sales of antibiotics

Finding on who frequently demand OTC sales of antibiotics shows that customers come from all segment of the population. However, it was found that often the poor, less educated and older age groups seek nonprescription sales of antibiotics. This was underscored by one of the participant that:

*“We encounter all population categories who seek to get antibiotics without prescription. Nevertheless, the older and less educated ones are those who frequently demand nonprescription sales of antibiotics. Such customers assume their demand of OTC dispensing of antibiotics is normal (CMP001).”*

Contrary to this, one of the participants stated that the educated customers are the ones who were frequently demand for nonprescription sales of antibiotics. He went on stating that *“educated customers come with information on their problem and medicines and at the pharmacy they request us for specific antibiotics (CMP004).”* Some participants (*n* = 2) mentioned that the younger age group are the ones who frequently go to pharmacy for antibiotics without prescription. Observations at the five selected pharmacies also revealed that relatively younger age groups of the population were found to seek antibiotics without presenting prescription.

### Antibiotics frequently sold without prescription

Finding shows that Amoxicillin, Ciprofloxacin and Cotrimoxazole were the most common antibiotics sold in community pharmacies without prescription. These drugs were identified for presumed treatment of diarrhea, common cold, tonsillitis and other respiratory infections. One of the participants stated that:

*“….these times, Amoxicillin is a famous medicine and everybody including children are coming to buy this medicine. My experience showed me that Amoxicillin is the most commonly nonprescription sold antibiotics here. Nonprescription sale of Ciprofloxacin is also becoming common. You know, it is worrisome that the future of these antibiotics and others is daunting given customers tend to get medicine without prescription and professionals continue to sell such medicines (CMP005).”*

Similarly, during the time of observation, many customers were buying Amoxicillin and Ciprofloxacin without prescription. In almost all of the cases pharmacy professionals were dispensing those medicines without valid prescription.

### Reasons for over the counter sale of antibiotics

All participants argued that the situation of OTC dispensing practice of antibiotic in Addis Ababa was becoming common and different reasons were identified for such practices. Four major reasons were identified to explain OTC sale of antibiotics. These were attributed to customers, owners of pharmacies, professionals’ interest and regulatory mechanism.

### Customers’ demand

It was found that continued customers’ demand is a major reason for maintaining OTC sale of antibiotics. The study participants reported that factors related to customers’ previous experiences, lack of awareness about risks of nonprescription use of antibiotics, prolonged waiting time at the health facilities to get prescription, unnecessary and high costs of physician visits were frequently mentioned reasons by their customers to demand antibiotics without prescription. When pharmacy professionals advise customers to visit health facilities, majority of the respondents (*n* = 4) stated that customers mostly reacted negatively. This was strengthened by one of the participants that:

*“…customers who relish benefits from taking antibiotic usually wanted to get the same antibiotic without prescription. If we refuse to dispense and advise them to go to clinics, most of them are not willing to do so. Longer waiting time to get prescription in public health facilities and higher cost of visit in private clinics are most of the complaints of the customer (CMP003).”*

Physician visits for diagnosis was considered unnecessary if customer is familiar with the symptoms and previous experience of getting relief by taking the antibiotics s/he is requesting for. It was gathered that if pharmacy professionals refuse to sale antibiotics without prescription, customer would easily get it from another pharmacy and the professional who insist not to dispense without prescription would loss his/her customers ultimately affecting the business. Thus, majority (*n* = 4) of participants contend that it is both not easy and counterproductive to convince customers to produce prescription to buy antibiotics. This was further substantiated that:

*“……customers believed that going to clinic have additional cost that they prefer to buy antibiotics from pharmacy without prescription. If you are not willing to sale without prescription, some of them confront you and would get the medicine from other pharmacy without prescription anyway. Besides, professionals are indirectly obliged to dispense antibiotics without prescription since they don’t want to lose their job (CMP005).”*

Another participant strengthened this:

*“I was one of the opponents of OTC dispensing of antibiotics. When I refuse to do so, many of my customers were telling me that they will get it from other pharmacy. They always tell me either to sale antibiotics without prescription to increase my profit or leave the business. I think it seems they were right and to be honest, now I am dispensing antibiotics without prescription (CMP001).”*

The behavior of prescriber was also influencing the customer behavior and one of the participants mentioned that even some of their customers were arguing them not to visit the health facilities because in the previous time they repeatedly obtained the same antibiotics. One participant stated this*” “….some of the customers complained to me that even they go to the clinics, they always obtained the same prescription like Augmentin (Amoxicillin clavulanate) as mentioned by most of my customers. As a result, most of the customers questioned the necessity of going to clinic as they expect same antibiotic will be prescribed again and hence they opted to buy the same antibiotic directly from the pharmacy (CMP004).”*

### Owner’s expectation

Findings related to owners expectation shows that majority (*n* = 3) of participants raised owners influence as one of the reasons for increasing OTC sales of antibiotics. In this regard, most of the owners expect professionals to dispense medicines without prescription. One of the respondents stated that *“….if we don’t dispense antibiotics without prescription, there is pressures from both sides* i.e. *the customer needs medicines and owners expect us to sale more no matter how (CMP004).”*

Some (*n* = 2) of the professionals also commented that even if one pharmacy insist not to sell medicine without prescription, nearby pharmacies as well as clinics would so contributing to OTC sales of antibiotics. This has been stated by one participant that *“…you can see many clinics around here that I guess are selling medicines although they are not allowed to do so. I am saying this because I haven’t received prescriptions from them whatsoever. So, you can imagine how difficult it would for the owner even to pay for house rent unless I sale antibiotics in any ways possible (CMP003).”*

### Professionals’ conflict of interest

Pharmacy professionals who participated in the study were all employed by owners. All participants (*n* = 5) pointed out that making financial profit is basic expectation from owners. As such every professional intends to draw as many customers as possible to maximize profit and maintain position. Consequently, majority (*n* = 4) of respondents pointed out to ignore their professional code of ethics. This was stressed that:

*“…….for someone in business, s/he needs to satisfy the customer. These days the purpose of dispensing is becoming solely to retain the customers and maximize profit. That is the expectation from the customers as well as the owners (CMP001).”*

All participants, however, reflected that nonprescription sale of antibiotic is wrong practice. One participated strengthened this: *“every professional knows nonprescription sale of antibiotics is illegal and unethical practice. But the practice continues as professionals’ main interest is to maximize their profit (CMP002).”.”*

### Weak regulatory mechanism

Finding shows that weak regulatory mechanism to enforce implementation of policies restricting nonprescription sale of antibiotics was mentioned by all (*n* = 5) respondents as one of the major reasons for nonprescription sales of antibiotics. All respondents indicated that the unethical dispensing of antibiotics without prescription is not because of lack of knowledge or awareness about its consequences but due lack of strong regulatory enforcement mechanism. Participants agree that customer’s demand for antibiotics without prescription and owner’s interest to reap more profit is reinforce by lack of functioning regulatory mechanism. One of the participants argued that:

*“….these time we can say there is no regulation of antibiotics and I think the weak regulation system is the main reason for continued and expanding sale of antibiotics without prescription. But I don’t think there is knowledge gap on consequence of antibiotic misuse among professionals including druggists. Unlike antibiotics, Narcotic and Psychotropic substances for example are not sold without prescription. I will never dispense it to you at any cost for fear of serious cost me to the extent of getting my license revoked and closure of the pharmacy (CMP002).”*

All participants called for strong regulatory mechanism to control nonprescription sale of antibiotics and limit its associated consequences. All participants suggested to strictly forbidden nonprescription sales of antibiotics and penalize violators as emphasized by one of the participants:

*“…..Generally, in our country we respect laws instead of moral authority. So, the Regional Health Bureau, FMHACA and other concerned bodies should consider strictly prohibiting nonprescription dispensing of antibiotics (CMP002).”*

Another participant also stated that:

*“…..to me putting regulatory mechanism would help. We know antibiotics are prescription only medicine and OTC sale of antibiotics is a violation of the regulatory standards. Hence, strong enforcement just like Narcotic and Psychotropic substance is needed (CMP003).”*

Although majority (*n* = 3) of participants don’t feel professionals’ have knowledge problem about the consequences of antibiotic misuse, they insisted the need to continuously update on the extent and burden of the problem in connection to nonprescription sales of antibiotics. One of the participants stressed that: *“professionals may know the consequence of nonprescription dispensing of antibiotic. But, I don’t think they are well aware of its burden. Thus, repeated educational campaigns should be organized to show them its level of health and economic impacts which then can contribute to decreasing OTC sales antibiotics (CMP005).”*

## Discussions

The study revealed that nonprescription sale of antibiotics in Addis Ababa is getting common. The study participants have admitted that despite knowing it is against the law, selling antibiotics as OTC medicines is common. It is documented that such nonprescription dispensing practice often leads to a wrong choice and/or dispensing insufficient doses to customers with little history taking and inadequate counseling [19, 26]. This could lead to life threatening adverse events and masking of underling infectious disease which otherwise could have been easily identified and treated at early stage [32]. Morgan et al. [20] also described the nonprescription sales of antibiotics as dangerous practice as it is one of the main factors accelerating the emergence of antibiotic resistance [20]. Since the nonprescription sale of antibiotics is now a universal problem, as it is the case from this study at least in Addis Ababa, it is suggested to be approached from a global perspective [19].

The causes of nonprescription sales of antibiotics may vary from country to country due to different underlying contexts. A study from Southern Europe showed that weak regulation of antibiotics was the most common reason for nonprescription sales of antibiotics. Similar to the finding from this particular study, the common reasons for breaking the law include: everyone was doing it, if they didn’t satisfy customers then the nearest rival pharmacy would and the interest to maximize profit [20]. Furthermore, customer’s interest to buy antibiotics without prescription was desirable since bypassing physicians was believed to be cheaper as there is no cost other than the cost of medicine. In general, an in-depth analysis of the study indicated that the owners interest to maximize revenue, professional interest to keep their job and customer need together with weak regulatory enforcement mechanism were the critical reasons for increasing OTC sale of antibiotics. Evidences reveal that many pharmacy professionals’ are interested to work as businessperson which forces them to disregard their professional responsibility instead of considering themselves as member of the healthcare team [21]. Another study done by *Radyowijati and Haak* also mentioned that the desire to meet customer demand, economic incentives from selling more antibiotics, professionals’ lack of appropriate knowledge and awareness about rational use of antibiotics, marketing influences, lack of regulation and enforcement were mentioned as the major reasons for OTC sale of antibiotics [33]. In this study, combination factors at the level of buyers, owners, sellers and regulation were found to maintain and exacerbate the potential dangers of taking medicines without prescription in Addis Ababa.

Controlling OTC sale of antibiotics through different mechanisms is an important to combat bacterial resistance and other related problems [23]. In this study, participants proposed on how to control the nonprescription sales of antibiotics. Strict regulatory enforcement mechanism and organizing awareness creation campaigns were suggested as most effective strategies. Although interventions are often context specific, the strict regulatory enforcement and organizing awareness campaigns with special emphasis to the general public were also recognized as the most operative intervention tools elsewhere [23, 34]. This has been evidenced by a study done in Chile and South Korea where the regulatory measures have shown great impact on controlling nonprescription sale of antibiotics and have improved resistance profiles of diseases [35, 36].

The present study also revealed that Amoxacillin, Cotrimoxazole and Ciprofloxacin were the most common of the antibiotics that are dispensed by pharmacy professionals to treat complaints such as diarrhea, common cold, tonsillitis and other respiratory infections. These types of antibiotics were found to be similar to the quantitative findings in different countries but in some of the countries there were little differences in the disease conditions these antibiotics are used for [19, 20]. Some of this variation might be ascribed to variations in the clinical scenario presented to the professionals. Such dispensing of antibiotics for non-bacterial diseases which actually don’t require antibiotics could be catastrophic [17].

## Conclusions

The study participants disclosed that OTC sale of antibiotics was a common practice in the community pharmacies of Addis Ababa. Weak regulatory enforcement mechanism, professional conflicts of interest, owner’s influence, customer’s demand and prescriber were the main reasons for continued sales of antibiotics without valid prescription. Thus, strict regulatory enforcement and structured educational campaigns for the public are suggested to control or limit the sales of antibiotics without prescription. Furthermore, detailed studies are recommended to figure-out the magnitude of the problem, what proportions of the community are affect and what are the triggering factors.

## Abbreviations

FMHACA: Food, Medicine, and Health Care Administration and Control Authority
OTC: over the counter
WHO: World Health Organization
